# Maternal Stress Potentiates the Effect of an Inflammatory Diet in Pregnancy on Maternal Concentrations of Tumor Necrosis Factor Alpha

**DOI:** 10.3390/nu10091252

**Published:** 2018-09-06

**Authors:** Karen L. Lindsay, Claudia Buss, Pathik D. Wadhwa, Sonja Entringer

**Affiliations:** 1 Departments of Pediatrics, University of California, Irvine, CA 92697, USA; kllindsa@uci.edu; 2 UC Irvine Development, Health and Disease Research Program, University of California, Irvine, CA 92697, USA; claudia.buss@charite.de (C.B.); pwadhwa@uci.edu (P.D.W.); 3 Charité–Universitätsmedizin Berlin, Corporate Member of Freie Universität Berlin, Humboldt-Universität zu Berlin, and Berlin Institute of Health (BIH), Institute of Medical Psychology, Charitéplatz 1, 10117 Berlin, Germany; 4 Departments of Psychiatry & Human Behavior, and Obstetrics & Gynecology, University of California, Irvine, CA 92697, USA

**Keywords:** dietary inflammatory index, inflammation, pregnancy, prenatal diet, prenatal stress, tumor necrosis factor-alpha

## Abstract

Maternal inflammation during pregnancy is known to adversely impact fetal development, birth outcomes, and offspring physical and mental health. Diet and stress have been identified as important determinants of inflammation, yet their combined effects have not been examined in the context of pregnancy. The aim of this study was to examine the relationship between maternal diet with inflammatory potential and psychological stress, and to determine their interaction effect on concentrations of tumor necrosis factor (TNF)-α across pregnancy. We conducted a prospective longitudinal study of *n* = 202 women with three assessments during pregnancy, which included: ecological momentary assessment (EMA) of maternal stress using the perceived stress scale (PSS) short version; 24-h dietary recalls from which the dietary inflammatory index (DII) was computed; and serum measurements of TNF-α. Across pregnancy, higher perceived stress was associated with consumption of a more pro-inflammatory diet (*r* = 0.137; *p* < 0.05). In a linear regression model adjusted for covariates, DII was positively associated with TNF-α (*B* = 0.093, *p* = 0.010). The effect of the pro-inflammatory diet on concentrations of TNF-α was more pronounced in women reporting higher levels of stress (*B* = 0.134, *p* = 0.018 for DII*PSS interaction). These results highlight the need to consider nutrition and stress concurrently in the context of inflammation during pregnancy.

## 1. Introduction

Embryonic and fetal life represents a critical developmental period during which exposures to suboptimal conditions or insults can result in structural and functional changes in cells, tissues, and organ systems that independently—or through interactions with subsequent developmental conditions and environments—may confer critical long-term consequences for health and disease susceptibility [1,2,3,4,5]. In this context, maternal inflammation during pregnancy has been established as a key mediator of the effects of a diverse range of gestational conditions. A heightened pro-inflammatory milieu during pregnancy is associated with complications such as preeclampsia, adverse birth outcomes such as preterm birth [6,7,8,9], and subsequent susceptibility in the offspring for metabolic dysfunction and neurodevelopmental disorders [1,10,11,12,13,14]. Prenatal inflammation may influence fetal development via direct transfer of maternally-derived cytokines, programming the offspring immune system towards a Th-1 dominant pro-inflammatory state, and increasing the presence of brain inflammation [15,16]. Maternal inflammation may also exert indirect effects either by stimulating placental inflammation and subsequently fetal inflammation [17,18], or through placental modulation of nutrient transfer to the fetus, such as increased flux of glucose and lipids, which, in turn, may program longer-term susceptibility for obesity and metabolic dysfunction [11,19]. However, beyond the prevalence of infections and pre-existing chronic inflammatory conditions, there is a paucity of literature investigating the role of potentially modifiable factors on the pro-inflammatory milieu during pregnancy.

Two of the most extensively but independently studied factors that influence gestational biology and fetal programming of offspring disease risk are psychological stress and diet/nutritional status, each of which have the potential to influence inflammatory profiles among pregnant women [13,20,21,22,23,24,25,26,27]. It is well established that acute and chronic stress is associated with low-grade inflammation [28,29,30], including increased concentrations of tumor necrosis factor (TNF)-α [31,32,33], mediated by stress-related activation of the hypothalamic–pituitary–adrenal (HPA) axis and the autonomic nervous system. In terms of diet/nutritional status, emerging evidence suggests that different dietary patterns and specific dietary components may exert systemic pro- and anti-inflammatory effects [34,35,36,37,38,39]. Furthermore, evidence from studies of non-pregnant humans strongly supports the presence of a recursive, bi-directional relationship between nutrition and stress [40,41,42,43], such that stress can influence the quantity and quality of food consumed, as well as its metabolism, while diet can dampen or enhance the stress response at both a psychological and biological level.

Recent perspectives papers and reviews have advanced a strong rationale for considering the combined effects of diet and stress in pregnancy with respect to fetal programming of offspring health outcomes, including brain development, immunity, and obesity risk [43,44,45,46]. This is based on a combination of evidence from non-pregnant populations and animal models of pregnancy, wherein an interactive effect of diet and stress has been found to affect metabolic [47,48,49] and inflammatory [50,51,52] pathways. While a handful of human pregnancy studies have reported associations between maternal psychosocial state and diet/nutritional status [53,54,55,56,57,58,59], there are, to the best of our knowledge, no published studies that have investigated the interactive effects of maternal diet and stress on the inflammatory milieu during pregnancy.

From among the various approaches to classify and quantify dietary patterns or overall diet quality, the dietary inflammatory index (DII), developed by Shivappa et al. [60], is a population-based measure of the inflammatory potential of an individual’s diet. The DII has been used in numerous studies and has been shown to be related to metabolic syndrome and cardiovascular disease [61,62,63,64,65,66,67], asthma [68], and cancer [69,70,71,72,73]. In a cohort of non-pregnant women in the Nurse’s Health Study, the DII was found to significantly predict risk of depression, providing evidence for a link between diet and psychosocial states [74]. More recently, studies from four pregnancy cohorts have evaluated the clinical and inflammatory effects of DII [25,75,76,77]. Three of the four studies reported positive associations between DII and biomarkers of maternal inflammation [75,76,77] and two reported that maternal DII may predict neonatal and child adiposity [76,77]. However, to date, the association between DII and maternal psychosocial state, or the combined effects of the DII and psychosocial state on inflammation in pregnancy, have not been examined.

Reliable characterization of maternal stress also poses challenges. The majority of human studies on the effects of stress during pregnancy have relied almost exclusively on self-reported retrospective-recall measures of stress [21], which are prone to numerous systematic biases that undermine their validity. However, recent technological and methodological advances now afford the opportunity to apply ecological momentary assessment (EMA) approaches to the study of maternal stress in human pregnancy. EMA methods emphasize the longitudinal, repeated collection of information about respondents’ momentary or current state and affect in real time and ecologically valid naturalistic settings. Immediate reports of a respondent’s current state or activity do not require retrieval from memory and other processing, and are accordingly less subject to distortions and biases. EMA techniques also require that assessments are timed carefully to span a wide range of times and locations, therefore tapping at the respondent’s full repertoire of states and behaviors.

Our study focuses on the pro-inflammatory cytokine TNF-α as a key outcome of interest because it is a potent, multifunctional cytokine that plays pivotal role in autocrine and paracrine processes during reproduction, including placental differentiation, embryogenesis, and parturition. TNF-α is secreted from type 1 T helper (Th1)-maternal and placental cells as part of the cell-mediated immune response, and it has both apoptotic and anti-apoptotic properties, depending on its receptor expression and activity [78]. Indeed, elevated levels of TNF-α in early gestation may be embryotoxic, leading to miscarriage and embryo maldevelopment [79,80]. Although advancing gestation is associated with a shift toward the Th-2 immune response and greater secretion of anti-inflammatory cytokines, previous longitudinal studies of uncomplicated pregnancies have shown that TNF-α increases as pregnancy progresses [6,81,82,83]. In pregnancies complicated by obstetric risk conditions, sustained elevated levels of TNF-α in mid-late pregnancy is associated with insulin resistance [81], preeclampsia [84,85,86] and premature rupture of membranes [6].

**Figure 1:**
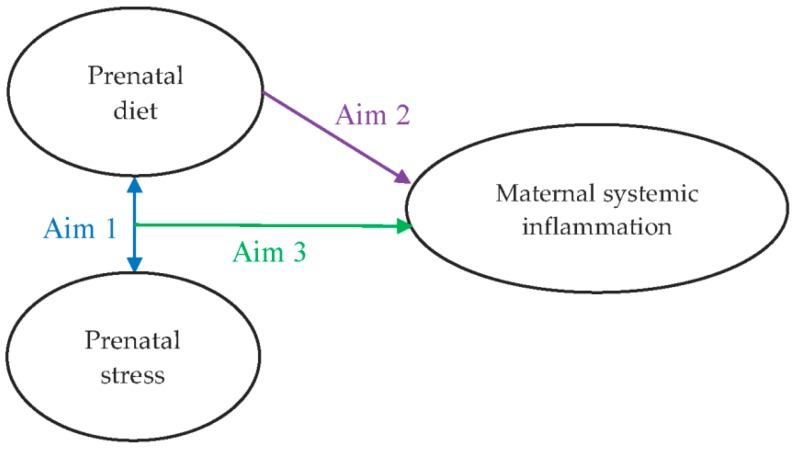
Hypothesized interaction between maternal prenatal diet and stress and the influence on systemic inflammatory profile in pregnancy.

The aims of this study were to examine: (1) the bivariate relationship between stress and dietary patterns during pregnancy; (2) the independent effect of a diet with inflammatory potential during pregnancy on maternal TNF-α levels during pregnancy; and (3) the moderating effect of maternal stress on the hypothesized link between maternal diet and inflammation (Figure 1). 

## 2. Materials and Methods

### 2.1. Participants

Healthy pregnant women were recruited for a longitudinal, prospective cohort study at the University of California, Irvine (UCI), Development, Health and Disease Research Program. The study was approved by the UCI Institutional Review Board and written, informed consent was obtained from all participants. 

**Figure 2:**
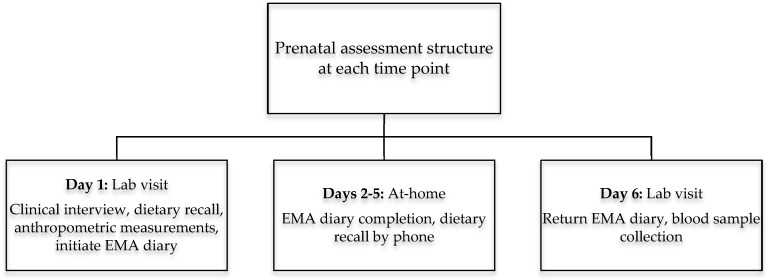
Structure of prenatal assessments in early (10–12 weeks), middle (20–22 weeks), and late (30–32 weeks) pregnancy.

Women were eligible for inclusion if they were >18 years of age with a singleton, intrauterine pregnancy, and non-diabetic. Women were recruited in the first or early second trimester from obstetric clinics at UC Irvine Medical Center in Orange, California, and affiliated obstetric clinics in surrounding areas. Participants completed up to three assessments at a mean ± standard deviation gestational age of 12.9 ± 1.7, 20.5 ± 1.4, and 30.4 ± 1.4 weeks. Each assessment involved two visits to the laboratory, four days apart, as depicted in Figure 2. The study sample of *n* = 202 represents the group with complete EMA, dietary, and TNF-α data.

### 2.2. Sociodemographic and Clinical Data

On the first visit at each assessment, demographic and clinical data were obtained via structured interview. Maternal socioeconomic status (SES) was defined as a combination (mean) of maternal educational level (originally assessed in categories from less than high school to advanced degree (master/doctorate) and then recoded into values from 1 to 5) and household income (originally assessed in categories from ≤ $15,000 to ≥ $100,000 and then recoded into values from 1 to 5). Women were asked about any occurrence of obstetric complications during interviews at each assessment, which was further verified from the medical record at the end of pregnancy. Pregnancies were characterized as ‘obstetric risk’ (dummy variable, ‘yes’ or ’no’ for presence of obstetric risk during pregnancy), if at least one of the following conditions were confirmed: (a) gestational diabetes; (b) preeclampsia or hypertension; (c) anemia; (d) severe infection (e.g., cytomegalovirus, toxoplasmosis, rubella, varicella, mycoplasma, or any sexually transmitted infection); and (e) vaginal bleeding that was present in both middle and late pregnancy (early pregnancy vaginal bleeding was excluded). Pre-pregnancy body mass index (BMI) was computed from self-reported pre-pregnancy weight and measured height (to the nearest 0.1 cm) using a stadiometer, according to the formula kg/m^2^.

### 2.3. Ecological Momentary Assessment (EMA) Protocol to Assess Stress

At the beginning of each prenatal assessment, women were provided with a smartphone programmed with a short questionnaire to assess their emotional and psychological state under ambulatory, naturalistic settings. Each assessment period included two-work days and two weekend days. Diary entries were preprogrammed to signal the participant an average of one time per hour (i.e., every 60 min ± 10 min.) during waking hours of the four-day monitoring sequence reflecting a time-based, stratified random sampling design. Each EMA diary entry included a 4-item short version of the original 10-item perceived stress scale (PSS) [87], which asked the participant to rate on a scale from 0–4 how they currently felt with respect to the following: (i) in control; (ii) confident; (iii) things were going their way; and (iv) overwhelmed. Items (i) to (iii) were reverse coded and then all responses were summed to produce a PSS score for each diary entry, with a higher score indicating greater perceived stress levels. The mean PSS score from all diary entries completed over each four-day assessment period was then computed. Mean PSS scores from each assessment period were significantly correlated across pregnancy time points (early and mid-pregnancy *r* = 0.763, *p* < 0.001; mid- and late-pregnancy *r* = 0.823, *p* < 0.001), and thus an overall mean pregnancy value across all three assessments was computed.

### 2.4. Dietary Assessments

Dietary intakes were assessed via three interviewer-administered 24-h dietary recalls using the multiple-pass method at the time of each prenatal assessment period. On the first laboratory visit at each assessment, a trained nutritionist conducted the first dietary recall in person with participants, followed by two further dietary recalls by telephone on non-consecutive days, within two weeks of the initial interview. Dietary intake data were entered to the Nutrition Data Software for Research (NDSR) program to generate daily nutrient and food group intake variables, which were averaged over the three dietary recall days at each assessment period. The DII was computed from this data as previously described [60]. Briefly, pre-defined dietary intake variables that are associated with inflammatory states were first converted to *z*-scores by subtracting the global-standardized mean value from each dietary variable and dividing by its global standard deviation value. The *z*-score variables were converted to percentiles, centered around 0, and then each centered percentile value for each food parameter was multiplied by its respective ‘inflammatory effect score’ to obtain the food parameter specific DII score. Individual food parameter scores were then summed to produce a total DII score per participant per assessment period. A positive DII value indicates a pro-inflammatory diet, while a negative score indicates an anti-inflammatory diet. The DII was originally developed based on 45 different food parameters, although it is rare that all of these parameters are available in any given dataset. The 24-h dietary recall data analyzed in NDSR in the present study includes 32 food parameters which were used to compute the DII score: energy, protein, total fat, saturated fat, monounsaturated fat, polyunsaturated fat, omenga-3 fatty acids, omega-6 fatty acids, trans fatty acids, cholesterol, carbohydrate, dietary fiber, alcohol, vitamins A, B1, B2, B3 (niacin), B6, B9 (folate), B12, C, D and E, beta-carotene, iron, magnesium, zinc, selenium, caffeine, green/black tea, garlic, and onion. DII values were significantly correlated across pregnancy time points (early and mid-pregnancy *r* = 0.435, *p* < 0.001; mid- and late-pregnancy *r* = 0.367, *p* < 0.001) and the mean pregnancy value was computed and used for analyses.

### 2.5. Tumor Necrosis Factor-alpha (TNF-α)

On the second visit to the research laboratory during each assessment period, maternal antecubital venous blood samples were collected in serum tubes (BD Vacutainer, New Jersey, USA). Serum samples were allowed to clot for 30 min at room temperature and were centrifuged at 4 °C at 1500× *g*. Serum was then separated and stored at −80 °C. TNF-α was determined by electrochemoluminescent immunoassay on a Meso Scale Discovery (MSD) instrument (SECTOR Imager 2400; Gaithersburg, MD, USA) in batches, with a coefficient of variability <10% and the lower limit of detection was 0.04 pg/mL. TNF-α data were assessed for normality through inspection of histograms and each were found to have a non-normal distribution. Outliers were handled through winsorization to the point of three standard deviations above the mean (*n* = 4 outliers detected) followed by log-transformation. As TNF-α values were significantly correlated across assessment time points (early and mid-pregnancy *r* = 0.652, *p* < 0.001; mid- and late-pregnancy *r* = 0.530, *p* < 0.001), the mean pregnancy value was computed to represent the total TNF-α production across gestation and used in statistical analyses.

### 2.6. Statistical Analyses

Descriptive statistics were used to describe maternal characteristics and the distributions of the DII, PSS, and TNF-α levels of the population at each assessment period and as mean values across pregnancy. Repeated measure ANOVA was conducted to test whether there were significant changes across pregnancy assessments for each of DII, PSS, and TNF-α. Bivariate associations between mean pregnancy DII, PSS, TNF-α and maternal characteristics were analyzed by Pearson’s correlations or Spearman rho correlation for continuous (maternal age, parity, SES index, pre-pregnancy BMI) or categorical (ethnicity, obstetric risk, smoking) data, respectively. The association between DII and PSS as continuous variables was graphically depicted in a bar plot, whereby PSS was dichotomized as low vs. high stress using the median value. Those maternal characteristics that were found to significantly correlate with the predictor (mean pregnancy DII and/or PSS) or outcome (mean pregnancy TNF-α) variables were considered as covariates in regression analyses. Linear regression was first used to test the main effect of mean pregnancy DII (independent variable) on TNF-α, with and without adjusting for confounding factors (pre-pregnancy BMI, SES index, obstetric risk category, ethnicity). Variance inflation factor (VIF) and tolerance statistics were used to assess the degree of multicollinearity across independent variables. The product of the mean pregnancy DII and PSS scores was computed to generate the DII*PSS interaction term. The linear regression models as described above were then repeated to include this interaction term as well as the mean pregnancy DII and PSS scores as independent variables. 

Lastly, since the presence of severe infection could have exerted an acute effect on TNF-α levels, a sensitivity analysis was performed by repeating the above regression models with TNF-α values excluded for those women in whom severe infection was reported (*n* = 15). All statistical analyses were performed with SPSS for Macintosh, version 24.0 (SPSS Inc., Chicago, IL, USA) and results were considered statistically significant at the level of *p* < 0.05.

## 3. Results

Descriptives of maternal sociodemographic factors and the mean values for DII, PSS, and TNF-α at each assessment time point and across pregnancy are presented in Table 1. Mean pregnancy DII ranged from −4.29 (most anti-inflammatory diet) to +3.68 (most pro-inflammatory diet) with an average score of −0.0003, mean pregnancy PSS ranged from 0.01 (no stress) to 2.46 (most stressed) with an average score of 1.05, and mean pregnancy TNF-α ranged from 1.15 to 19.13 pg/mL with an average value of 8.65 pg/mL. DII decreased with advancing gestation (*p* = 0.023), indicating a shift towards a more anti-inflammatory diet in later pregnancy, PSS remained stable across pregnancy (*p* = 0.216), while TNF-α showed the expected increased from early to late pregnancy (*p* = 0.054). 

**Figure 3:**
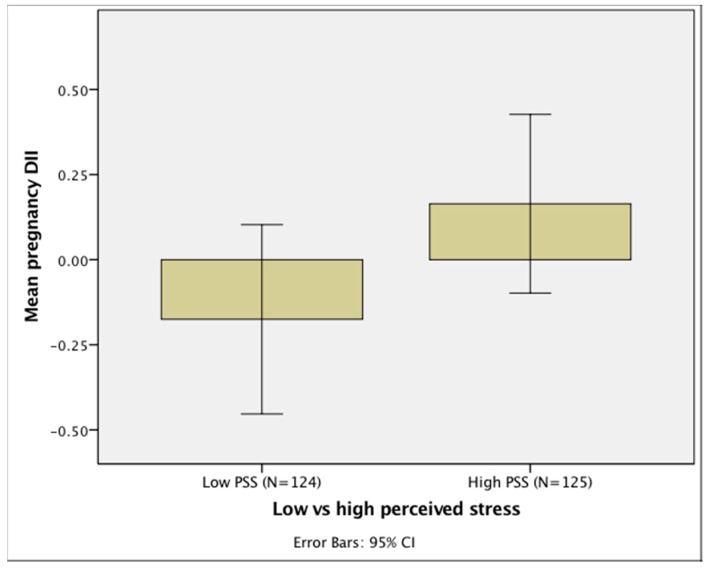
Bar chart depicting the association of prenatal DII score with maternal PSS. CI: Confidence Interval. Caption: DII, dietary inflammatory index; PSS, perceived stress score. High and low PSS is operationalized as mean pregnancy PSS score above and below the median value. The DII score of the maternal diet is higher among women with a higher PSS score, indicating consumption of a more pro-inflammatory diet among women experiencing higher stress levels. PSS and DII as continuous variables were correlated in statistical analyses.

The bivariate analyses presented in Table 2 show that mean pregnancy DII was significantly positively correlated with PSS (*r* = 0.137, *p* = 0.034). Thus, women who reported higher levels of perceived stress consumed a diet of higher inflammatory potential (see Figure 3). DII also was positively correlated with TNF-α (*r* = 0.195, *p* = 0.006), but PSS was not correlated with TNF-α (*p* = 0.556). SES index was significantly inversely associated with mean pregnancy DII (*r* = −0.264, *p* < 0.001) and PSS (*r* = −0.210, *p* = 0.001), such that women of a higher SES consumed a diet with lower inflammatory potential and reported lower perceived stress. Pre-pregnancy BMI was significantly positively associated with DII (*r* = 0.262, *p* < 0.001), such that women with a higher BMI before pregnancy consumed a more pro-inflammatory diet across pregnancy. Mean pregnancy PSS was significantly correlated with obstetric risk (*r* = 0.142, *p* = 0.026) and ethnicity (*r* = −0.138, *p* = 0.036), such that Hispanic women and those with an at-risk pregnancy reported higher PSS scores.

Linear regression models were run unadjusted and adjusted for SES index, pre-pregnancy BMI, presence of obstetric risk, and ethnicity. The results of the linear regression models testing the main effect of DII and the effect of the DII*PSS interaction on TNF-α are presented in Table 3 and Table 4, respectively. Mean pregnancy DII was significantly positively associated with TNF-α across pregnancy (*B* = 0.084, *p* < 0.001), which remained significant after adjusting for covariates (*B* = 0.093, *p* = 0.01) (Table 3), suggesting that consumption of a prenatal diet with greater inflammatory potential is associated with higher concentrations of TNF-α. The VIF is low (<1.5) and the tolerance statistic ranges from 0.65–0.93 across independent variables in the adjusted model, indicating that the results are not influenced by multicollinearity. 

**Figure 4:**
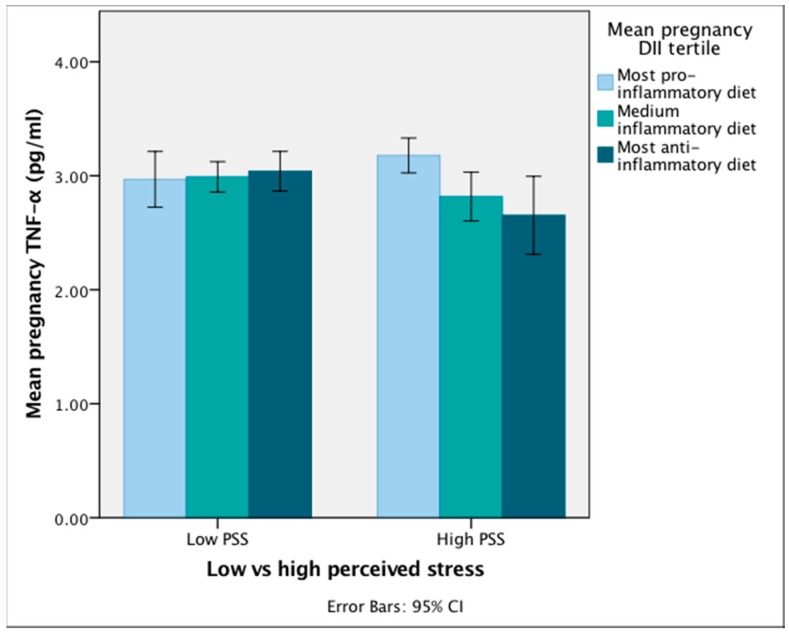
Mean pregnancy TNF-α levels among pregnant women with high and low perceived stress scores, according to DII tertile. CI: Confidence Interval. Caption: DII, dietary inflammatory index; PSS, perceived stress score; TNF, tumor necrosis factor. Although DII, PSS, and TNF-α were entered to regression models as continuous variables, for the purpose of graphically depicting the effect of the DII*PSS interaction term on TNF-α, mean pregnancy DII was divided into tertiles and mean pregnancy PSS was dichotomized by the median value. A pro-inflammatory diet influences higher TNF-α levels only among women reporting higher perceived stress scores across pregnancy. Women who report lower perceived stress levels do not experience any difference in TNF-α levels, regardless of the inflammatory potential of their diet.

Furthermore, there was a significant association of the DII*PSS interaction term on mean pregnancy TNF-α, before and after adjustment for covariates (*B* = 0.127, *p* = 0.019 and *B* = 0.134, *p* = 0.018, respectively) (Table 4). Figure 4 graphically depicts the interaction, such that among women who report higher levels of perceived stress across pregnancy, TNF-α levels increased as the inflammatory potential of the diet increased. Meanwhile, among women with lower perceived stress levels, TNF-α was not associated with the DII score. In the regression models including the interaction term and PSS, the main effect for PSS was still not significant.

Since pre-pregnancy BMI could potentially be on the causal pathway of the effect of DII and the DII*PSS interaction on TNF-α levels, we also conducted the regression models without adjustment for BMI, but the afore-mentioned effects remained significant (main effect of mean pregnancy DII, adjusted for SES, obstetric risk, ethnicity: *B* = 0.105, *p* = 0.002; interactive effect of mean pregnancy DII*PSS, adjusted for SES, obstetric risk, ethnicity: *B* = 0.130, *p* = 0.022). This suggests that the effects of DII and the DII*PSS interaction on TNF-α levels persist across the full spectrum of pre-pregnancy BMI.

In the sensitivity analysis after excluding values for TNF-α when severe infection was reported, the main effect of mean pregnancy DII (*B* = 0.093, *p* = 0.004 unadjusted; *B* = 0.09, *p* = 0.014 adjusted), and of the DII*PSS interaction term (*B* = 0.131, *p* = 0.02 unadjusted; *B* = 0.134, *p* = 0.019 adjusted) on TNF-α remained significant.

## 4. Discussion

Results of the current study suggest that higher levels of perceived stress are significantly associated with consumption of a diet with greater inflammatory potential, and that perceived stress potentiates the effects of a pro-inflammatory diet on TNF-α levels across pregnancy. Since increased adiposity is strongly associated with low-grade chronic inflammation in non-pregnant and pregnant populations [88,89,90], including a positive association with TNF-α [91], we also explored the relationship of maternal pre-pregnancy BMI status with the effects of DII and the DII*PSS interaction on TNF-α across pregnancy. The results were not altered by either including or excluding pre-pregnancy BMI as a covariate, suggesting that a pro-inflammatory diet alone—as well as in combination with maternal stress—influences levels of maternal TNF-α irrespective of BMI status. While a handful of previous studies have examined the inflammatory profile and adverse clinical outcomes associated with a pro-inflammatory diet in pregnancy [25,75,76,77], to the best of our knowledge, this is the first study to demonstrate that the combination of maternal stress and poor diet interact to exacerbate the inflammatory milieu across gestation.

Maternal stress and depression have both been associated with higher circulating levels of IL-6 and TNF-α across pregnancy [27,92]. The present study did not find a significant correlation between maternal perceived stress scores and TNF-α, which is in alignment with findings from one other pregnancy cohort [93]. A study which performed an in vitro immune stimulation protocol found that greater subjective stress among women in the third trimester predicted an exaggerated cytokine production by lymphocytes [94], suggesting that maternal prenatal stress alters the inflammatory response to immune triggers. This may explain the results of the present study such that there was no main effect of perceived stress on TNF-α, yet in the presence of a habitual pro-inflammatory dietary pattern, greater maternal stress potentiated the effects of the diet on TNF-α.

Studies investigating the effects of prenatal dietary patterns associated with inflammation have reported mixed results. Neither a low glycemic index diet [95] nor an antihypertensive diet [96] among pregnant women exerted any effects on markers of inflammation, although a high-complex carbohydrate/low-fat prenatal diet was associated with lower expression of pro-inflammatory genes in adipose tissue [26]. The null findings with respect to inflammation in some of the aforementioned studies may be attributed to the characterization of diets, which may not necessarily address specific dietary components that have the potential to either induce or dampen an inflammatory response. The DII is particularly pertinent in this regard and has recently been evaluated in four other prenatal cohort studies, which have revealed mixed findings with respect to the effect of maternal DII on biomarkers of inflammation. Two of these studies reported that higher DII scores were associated with higher maternal plasma C-reactive protein (CRP) levels [75,76], which was the only inflammatory biomarker assessed, while a third study found that maternal DII was not associated with high sensitivity CRP but was positively associated with levels of Interleukin-6 [77]. The fourth study did not identify any significant associations between maternal DII and inflammatory cytokines [25]. Differential results across these previous studies and our study may be attributed to differences in sample size, cohort profile, and other methodological factors, including method of dietary intake assessment, timing and frequency of blood sample collections for measurement of inflammatory markers, and different assay platforms used. We also note that in the one previous pregnancy study that examined the relationship between DII and levels of TNF-α [25], the authors computed an alternative energy-adjusted version of the DII, which may explain differences in results from our study.

The observed association between maternal perceived stress and consumption of a more pro-inflammatory diet in this study is consistent with a large body of evidence from non-pregnancy studies that demonstrate a bi-directional relationship between dietary intakes and psychological stress [40,41,42,43]. Poor diet quality, with preponderance for consumption of unhealthy foods high in fat and sugar, is frequently reported across various cohorts in relation to perceived and/or experimental stress induction [97,98,99,100,101], the intake of which subsequently dampens the stress response, reduces feelings of anxiety and distress, and thus leads to a cycle of ‘emotional eating’ [48,102]. A handful of pregnancy studies have also reported associations between maternal psychosocial stress and unhealthy dietary patterns [53,59,103], suboptimal nutrient intakes [55,58], and disordered eating [54,56], yet none of these studies examined the effects of the diet-stress interactions on maternal gestational biology.

The biological mechanisms underlying the effects of diet on inflammatory profile is multifaceted, as various dietary constituents may exert pro- or anti-inflammatory effects via different mechanisms. For example, omega-3 long-chain polyunsaturated fatty acids are widely recognized for their anti-inflammatory properties, which may be attributed to their critical role in synthesizing eicosanoids, important lipid mediators that regulate inflammation and oxidative stress [104,105]. One mechanism by which vitamin D may regulate inflammation is through inhibition of the activity of the TNF-α converting enzyme, thus suppressing the release of TNF-α and related pro-oxidant markers [106]. On the other hand, a high sugar intake may induce hyperglycemia which is associated with spontaneous TNF-α secretion by peripheral monocytes due to downregulation of the CD33 membrane receptor that is responsible for inhibiting cytokine production [107]. The DII is designed to capture the full spectrum of such effects from the diet by classifying the inflammatory potential of an individual’s diet from a wide range of nutrients and non-nutritive dietary compounds [60]. An investigation of the contribution of individual DII components to the total DII score in our study population is beyond the scope of this paper, but we can speculate that the observed positive association between the DII score and TNF-α levels is attributed to a high consumption of processed foods, which are high in refined sugar and unhealthy fats, with a concomitant low intake of fruit and vegetables contributing to a poor micronutrient and anti-oxidant profile. One potential mechanism by which perceived stress moderates the effect of DII on TNF-α is via activation of the HPA-axis and glucocorticoid production, which stimulates gluconeogenesis and reduced insulin sensitivity to supply energy for the ‘fight or flight’ response in a stressed state [108]. If this state of stress-induced rise in blood glucose levels is accompanied by a habitual high intake of dietary sugar (which is a component of the DII), a low-grade state of hyperglycemia could enhance spontaneous TNF-α secretion from peripheral monocytes by the mechanism described above [107].

Strengths of this study include its longitudinal design and multiple assessments of maternal perceived stress and dietary intakes across gestation, rather than relying on a single assessment. Use of EMA methodology to assess psychosocial state in real-time and in free-living, naturalistic settings provides more reliable data compared to traditional retrospective questionnaire methods by reducing the risk of recall and saliency bias. Dietary intake assessments by multiple 24-h dietary recalls also generates detailed nutritional data for accurately computing the DII, which may be more reliable than the food frequency questionnaires for this purpose. However, as with all self-report dietary methods, there is an inherent recall bias, which may be a weakness of this study. The study cohort is diverse with respect to distribution of maternal SES, pre-pregnancy BMI and race/ethnicity, and thus representative of the general US population, although the smaller sample size compared to other recent populations examining DII in pregnancy may be a limitation. 

Our result of an interaction effect between diet and stress on the maternal inflammatory profile may have clinical significance, as elevated levels of TNF-α likely have consequences for fetal programming of disease risk. In the third trimester of normal pregnancy, and as reflected in the data from this cohort, there is an increase in placental production of TNF-α, much of which is released into the maternal circulation [81]. Elevated levels of TNF-α reduce insulin sensitivity in maternal organs, increasing the availability of circulating glucose and fatty acids that could be transferred to the fetus [109], thus predisposing to greater infant adiposity and later metabolic dysfunction. Under pro-inflammatory conditions such as maternal illness or infection, TNF-α signaling in the placenta via TNF receptor 1 is associated with fetal hypoxia and neuroproliferative defects in the fetal brain [110]. Therefore, identifying the potentially modifiable factors which influence inflammation in the mother during pregnancy present opportunities for primary prevention of adverse health outcomes in the offspring.

In conclusion, this study demonstrates that maternal stress and diet are significantly associated across pregnancy, and that a combination of these factors has the potential to exacerbate maternal levels of inflammation. Thus, these findings highlight the need to consider nutrition and stress concurrently in the context of pregnancy, although further research is required to investigate whether the interaction of prenatal diet and stress exerts long-term effects on offspring developmental and health outcomes.

## Figures and Tables

**Table 1:** Maternal sociodemographics, DII, PSS, and TNF-α levels across pregnancy.

Variable	Descriptive Statistics			
Age (years)	27.83 ± 5.40			
SES index	3.23 ± 0.96			
Pre-pregnancy BMI (kg/m^2^)	26.45 ± 6.35			
BMI category				
Underweight	9 (3.6)			
Normal weight	121 (47.8)			
Overweight	61 (24.1)			
Obese	53 (20.9)			
Hispanic ethnicity	107 (42.3)			
Smoking during pregnancy (yes)	20 (7.9)			
Obstetric risk pregnancy (yes)	51 (20.2)			
Parity				
0	103 (40.7)			
1	62 (24.5)			
2	49 (19.4)			
≥3	20 (7.9)			
	Early pregnancy	Mid pregnancy	Late pregnancy	Mean pregnancy
DII	0.261 ± 1.815	−0.100 ± 1.811	−0.202 ± 1.782	−0.003 ± 1.505
PSS	1.036 ± 0.581	1.043 ± 0.589	1.089 ± 0.599	1.046 ± 0.547
TNF-α (pg/mL)	8.219 ± 3.589	8.512 ± 3.753	9.216 ± 4.387	8.650 ± 3.236

Values presented as mean ± standard deviation or N (%) for continuous and categorical variables, respectively. BMI, body mass index; DII, dietary inflammatory index; OB risk, obstetric risk pregnancy; PSS, perceived stress scale; SES, socioeconomic status; TNF, tumor necrosis factor. SES index range is 1–5, computed as a composite of maternal highest level of education and total household income.

**Table 2:** Bivariate associations between TNF-α, DII, PSS, and maternal characteristics.

	Mean TNF-α	Mean DII	Mean PSS	Maternal Age	Parity	SES Index	Pre-Pregnancy BMI	Ethnicity	Obstetric Risk	Smoking
Mean TNF-α	1	0.195 **	−0.042	−0.002	−0.026	0.021	0.167 *	0.007	0.003	0.071
Mean DII	0.195 **	1	0.137 *	−0.118	0.121	−0.264 **	0.262 **	−0.075	0.022	0.012
Mean PSS	−0.042	0.137 *	1	−0.077	−0.045	−0.210 **	−0.139 *	0.138 *	0.142 *	0.019

Values represent correlation coefficients, computed by Pearson’s test for continuous variables and Spearman rho test for categorical variables (ethnicity, OB risk and smoking). * *p* < 0.05, ** *p* < 0.01. BMI, body mass index; DII, dietary inflammatory index; PSS, perceived stress scale; SES, socioeconomic status; TNF, tumor necrosis factor.

**Table 3:** Main effect of DII on TNF-α across pregnancy, unadjusted and adjusted for maternal covariates

Unadjusted Model (*R*^2^ Adjusted = 0.033)						Adjusted Model (*R*^2^ Adjusted = 0.043)						Collinearity Statistics	
Independent Variable	*B*	Std. Error	*p*	95% CI		Independent Variables	*B*	Std. Error	*p*	95% CI		Tolerance	VIF
Mean pregnancy DII	0.084	0.030	<0.001	0.025	0.143	Mean pregnancy DII	0.093	0.036	0.010	0.023	0.163	0.780	1.283
						SES index	0.064	0.059	0.280	−0.052	0.179	0.649	1.541
						Pre-pregnancy BMI	0.011	0.007	0.146	−0.004	0.026	0.750	1.332
						Ethnicity	−0.043	0.103	0.676	−0.247	0.161	0.929	1.077
						Obstetric risk	−0.108	0.110	0.326	−0.324	0.108	0.856	1.168

BMI, body mass index; CI, Confidence Interval; DII, dietary inflammatory index; SES, socioeconomic status; TNF, tumor necrosis factor; VIF, variance inflation factor.

**Table 4:** Interactive effect of DII*PSS on TNF-α across pregnancy, unadjusted and adjusted for maternal covariates

Unadjusted Model (*R*^2^ Adjusted = 0.055)						Adjusted Model (*R*^2^ Adjusted = 0.063)					
Independent Variables	*B*	Std. Error	*p*	95% CI		Independent Variables	*B*	Std. Error	*p*	95% CI	
Mean pregnancy DII	−0.038	0.061	0.539	−0.158	0.083	Mean pregnancy DII	−0.042	0.068	0.530	−0.176	0.091
Mean pregnancy PSS	−0.102	0.083	0.222	−0.265	0.062	Mean pregnancy PSS	−0.062	0.088	0.480	−0.236	0.111
Mean pregnancy DII*PSS	0.127	0.053	0.019	0.021	0.232	Mean pregnancy DII*PSS	0.134	0.056	0.018	0.023	0.245
						SES index	0.055	0.058	0.343	−0.060	0.170
						Pre-pregnancy BMI	0.012	0.008	0.123	−0.003	0.026
						Ethnicity	−0.015	0.104	0.886	−0.219	0.189
						Obstetric risk	−0.081	0.110	0.464	−0.298	0.137

BMI, body mass index; CI, Confidence Interval; DII, dietary inflammatory index; PSS, perceived stress scale; SES, socioeconomic status; TNF, tumor necrosis factor.
